# Ultrasound Performed by Emergency Physicians for Deep Vein Thrombosis: A Systematic Review

**DOI:** 10.5811/westjem.18125

**Published:** 2024-02-09

**Authors:** Daniel Hercz, Oren J. Mechanic, Marcia Varella, Francisco Fajardo, Robert L. Levine

**Affiliations:** * Jackson Memorial Hospital, Department of Emergency Medicine, Miami, Florida; † University of Miami, Department of Surgery, Miami, Florida; ‡ Mount Sinai Medical Center, Department of Emergency Medicine, Miami Beach, Florida; § Florida International University, Herbert Wertheim College of Medicine, Department of Medical Education, Miami, Florida; ∥ Florida International University, Herbert Wertheim College of Medicine, Department of Emergency Medicine and Critical Care, Miami, Florida

## Abstract

**Introduction:**

Point-of-care ultrasound (POCUS) performed by emergency physicians (EP) has emerged as an effective alternative to radiology department ultrasounds for the diagnosis of lower extremity deep vein thrombosis (DVT). Systematic reviews suggested good sensitivity and specificity overall for EP-performed POCUS for DVT diagnosis, yet high levels of heterogeneity were reported.

**Methods:**

In this systematic review and meta-analysis, we aimed to provide the most up-to-date estimates of the accuracy of EP-performed POCUS for diagnosis of DVT and to explore potential correlations with test performance. We performed systematic searches in MEDLINE and Embase for original, primary data articles from January 2012–June 2021 comparing the efficacy of POCUS performed by EPs to the local standard. Quality Assessment of Diagnostic Accuracy Studies-2 for individual articles are reported. We obtained summary measures of sensitivity, specificity, and their corresponding 95% confidence intervals (CI) using bivariate mixed-effects regression models. We performed meta-regression, subgroup, and sensitivity analyses as planned in the protocol CRD42021268799 submitted to PROSPERO.

**Results:**

Fifteen publications fit the inclusion criteria, totaling 2,511 examinations. Pooled sensitivity and specificity were 90% (95% CI 82%–95%) and 95% (CI 91%–97%), respectively. Subgroup analyses by EP experience found significantly better accuracy for exams performed by EP specialists (93%, CI 88%–97%) vs trainees (77%, CI 60%–94%). Specificity for EP specialists (97%, CI 94%–99%) was higher than for trainees (87%, CI 76%–99%, *P* = 0.01). Three-point compression ultrasound (CUS) was more sensitive than two-point CUS but was only statistically significant when limited to EP specialists (92% vs 88%, *P* = 0.07, and 95% vs 88%, *P* = 0.02, respectively).

**Conclusion:**

Point-of-care ultrasound performed by emergency physicians is sensitive and specific for the diagnosis of suspected DVT when performed by trained attending EPs. Three-point compression ultrasound examination may be more sensitive than two-point CUS.

## INTRODUCTION

Lower extremity deep venous thrombosis (DVT) is an acute medical condition that, if not urgently diagnosed and treated, can result in severe morbidity and mortality. Left untreated, the associated one-month mortality of acute DVT is 10–15%.^1^ Postphlebitic syndrome is seen in 23–67% of patients after resolution of the initial thrombosis.^2^ Further, DVT is a common problem representing up to 2% of diagnoses made in the emergency department (ED),^3^ ^,^ ^4^ making it a compelling “can’t-miss” urgent diagnosis. Compression ultrasonography (CUS) has become a widespread tool that makes the evaluation of DVT rapid and precise. Compression ultrasonography is recognized by the American College of Emergency Physicians and the American College of Radiologists as the standard of care for the diagnosis of DVT, supplanting older techniques.^5^ In addition to radiology department-performed CUS, point-of-care ultrasound (POCUS) performed in the ED has emerged as an effective diagnostic modality.^6^

The region of interest for most ED-based DVT POCUS protocols extends from the common femoral vein to the popliteal vein. Most DVT POCUS protocols include CUS of the common femoral vein, popliteal vein, and possibly the femoral vein.^7^ These are referred to as two-point or three-point CUS, respectively, depending on the number of sites interrogated. The clinical significance of isolated venous thrombosis of the calf is controversial; however, non-urgent outpatient surveillance is an accepted treatment.^8^ Finally, while isolated thrombosis of the iliac vein is a potentially life-threatening condition, it is rare and difficult to detect with existing sonographic techniques.^9^ Thus, distal DVT and isolated iliac vein thrombosis are not addressed in this review.

While ED-performed POCUS is accepted by emergency physicians (EP) and radiologists for the diagnosis of DVT, there exists substantial variability in the diagnostic accuracy of POCUS.^7^ Factors that may affect diagnostic accuracy include the experience and ability of the ultrasound operator, the number of anatomical sites of the lower extremity scanned,^10^ whether augmentation techniques are used (such as Doppler) and image interpretation (such as vessel identification and partial compressibility).^11^ ^,^ ^12^

Studies and reviews comparing the accuracy of ED-performed POCUS for the diagnosis of DVT to a radiology department-performed ultrasound span more than 20 years. Earlier studies were small, more likely based in the United States, and complicated by heterogeneous methods and results.^13^ Currently, to our knowledge, there exist no guidelines or best practices for ED-based DVT POCUS. With the last systematic review published almost a decade ago, we performed an updated systematic review to explore the diagnostic accuracy of ED-based POCUS compared to radiology department-performed ultrasound. We also explored factors affecting the diagnostic accuracy for the diagnosis of DVT through subgroup analysis and meta-regression of recent studies.

## METHODS

In this systematic review we aimed to assess the accuracy of bedside venous ultrasonography as performed by EPs when compared to those performed by the radiology department for the diagnosis of DVT of lower extremities in adult patients. The protocol for this review was accepted and registered on the International Prospective Register of Systematic Reviews (PROSPERO) under the number CRD42021268799.

### Search Strategy

We conducted a literature search in MEDLINE (via Ovid MEDLINE) and Embase (via Elsevier) for relevant, original studies published from January 2012–June 2021 to update from the latest published systematic review on the topic.^13^ The detailed list of search terms used is listed in the Appendix (supplemental material). We consulted with domain experts for unpublished studies and conducted a manual search of published literature from the references listed on the included articles. The language was restricted to English.

### Study Selection

Eligible studies were original, primary data, collected using cross-sectional and longitudinal study designs (cohort or randomized controlled trials), that included adult patients (age >18 years) presenting to the ED for which DVT was listed as a differential diagnosis and for which, as part of the diagnostic workup, an ultrasonographic exam was performed by an EP and an ultrasound was performed by the radiology department. A contrast venogram (angiography) was an acceptable alternative to a radiology department-performed ultrasound. We used the systematic review management tool, Covidence, for the screening of titles/abstracts and quality assessment of studies. At least two investigators (DH and MV or OH and MV) independently reviewed the titles and abstracts of the studies for eligibility.

Discrepancies in the eligibility decision were resolved by a third investigator (RL) after reviewing the full article. Reasons for exclusion were recorded. We excluded review articles, editorials or letters, expert opinions, comments, and animal experiments. Lastly, we excluded articles for which no information was available on the total number of true positives, true negatives, false positives, or false negatives.

### Data Extraction

At least two reviewers independently extracted data on the selected studies (DH and MV, or OM and MV). Collected information included the following: country where the study was performed; the type of US exam used for the index test (two-point or three-point); clinical experience (attending and/or trainee) and description of the formal training of physicians performing the index test; whether the original study had performed risk stratification of participants prior to the use of the index test; numbers of true positives, true negatives, false positives, and false negatives, sensitivity and specificity as reported; and corresponding measures of precision (confidence intervals [CI]). To assess potential biases in individual studies, we used the Quality Assessment of Diagnostic Accuracy Studies 2 (QUADAS-2) checklist. Disagreements were resolved by consensus, or by a third reviewer.

### Statistical Analyses

Summary measures of sensitivity, specificity, and their corresponding 95% CIs were obtained using bivariate mixed-effects regression models. We estimated I^2^ statistic assessing for study heterogeneity. In addition, inconsistencies were further explored through visual inspection of forest plots (for overlapping of sensitivity and specificity point estimates and corresponding 95% CI) and by subgroup analyses. Subgroup analyses, defined a priori, included stratification by the type of US study performed (two-point CUS vs three-point CUS); experience of physicians performing the index POCUS (completed specialty EM training or specialist/attending vs EM trainee or resident status); prevalence of DVT; sample size; risk of bias; and outlier status. We performed all analyses with STATA v16 (StataCorp LLC, College Station, TX).^14^

## RESULTS

**Figure 1.**
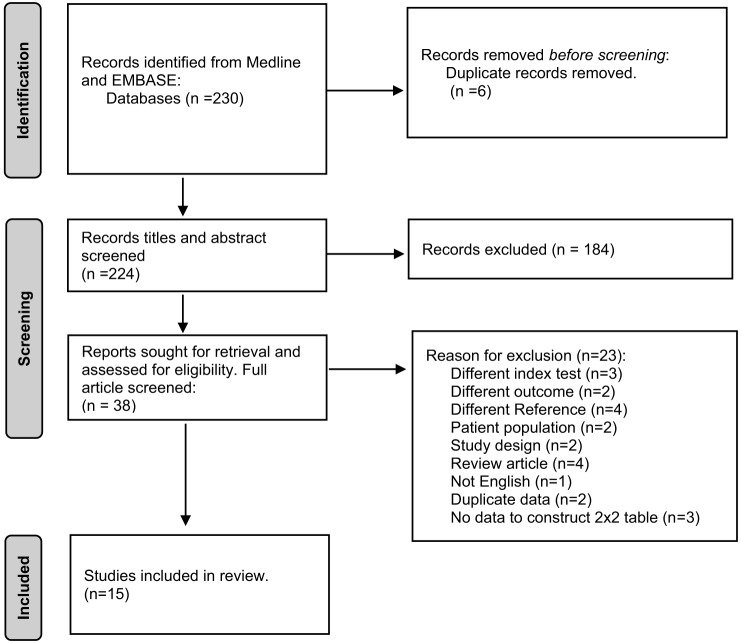
PRISMA flow diagram of the search and selection process for studies included in the meta-analysis.

We identified 230 studies in EMBASE and Medline that fit our search strategy (Figure 1). After removing duplicates, titles, and abstracts, we retrieved 38 studies for further evaluation based on inclusion criteria and abstract review. Fifteen publications^15^ ^–^ ^29^ remained after full text review with reasons for exclusion listed in Figure 1. Fourteen were full-length articles with one manuscript reporting two trials. One additional study reporting sufficient data for inclusion in the analysis was published as an abstract. In two instances, we obtained additional study characteristics via direct author correspondence.

### Characteristics of Studies

Studies varied greatly in geographic locations; three studies were done in the United States and Canada, two in Australia, and three in Iran, among other locations (Table 1). The number of diagnostic tests compared ranged from 56–385. Most studies reported data per patient, with two studies that reported results by limb.^15^ ^,^ ^18^ Prevalence of DVT in the samples varied from 10%–79%.^16^ ^,^ ^19^ ^,^ ^27^ About 50% of the studies used two-point ultrasound, and 50% used three-point. One publication tested both two-point and three-point US to the reference standard.^22^ Most studies used the locally available radiology department-performed DVT US as the reference standard. Lastly, pre-intervention training requirements for the EP operators varied greatly between studies, ranging from brief didactics to multi-day practical courses. Pre-existing experience was categorized as either completion of an emergency medicine (EM) postgraduate training program or by trainee status.

**Table 1.** Characteristics of studies selected for data abstraction.

Author, year	Patient’s country	Number of tests*	DVT prevalence %	Index test	Reference	Experience of physician performing the index test
Torres-Macho, 2012	Spain	76*	34	Two-point US	US done by radiologist	Attending
Abbasi, 2012	Iran	81	79	Three-point US (with Doppler)	Duplex US done by a 2^nd^-year radiology postgrad	EM resident
Crowhurst, 2013	Australia	178*	13	Three-point US	Duplex US done by radiologist (Doppler used if obese patient)	Attending
Poley, 2014	Canada	227	12	Two-point US	LC US done by radiologist or medical record review at 6 months in those who had no comprehensive LCUS	Attending + EM resident
Zitek, 2016	United States	385*	10	Two-point US	US done by radiologist	EM resident
Kim, 2016	United States	296	19	Three-point US (with Doppler)	LC US done by radiologist	Attending + EM resident
Pedraza-Garcia, 2017	Spain	109	54	Three-point US	US done by radiologist (with Doppler)	Attending
Zuker-Herman, 2018	Israel	195	26	Two- and three-point US	Duplex US done by radiologist	Attending + EM resident
Pujol, 2018	France	56	20	Two-point US	Duplex ultrasound done by a vascular certified practitioner.	Attending
Dehbozorgi, 2019	Iran	240	44	Three-point US	Duplex US done by radiologist	Attending + EM resident
Basaure, 2019	Chile	101	17	Three-point US	US done by radiologist with Doppler	Attending + EM resident
Jahanian, 2019	Iran	72	38	Three-point US (with Doppler)	US done by radiologist with Doppler	EM resident
Howland, 2019	Australia	100	10	Three-point US	Unclear	Attending
Elsenga, 2020	Netherlands	138	21	Two-point US (with Doppler)	rCUS done by radiologist	Attending + EM resident
Canakci, 2020	Turkey	266	26	Two-point US	US done by radiologist or venography	EM resident

Diagnostic assessment could be done per patients or per limb (*mark studies done per limb).

*DVT*, deep vein thrombosis; *US*, ultrasound; *ED*, emergency department; *LCUS*, limited compression ultrasound; *rCUS*, regional compression ultrasound; *EM*, emergency medicine.

### Primary Outcomes

**Figure 2.**
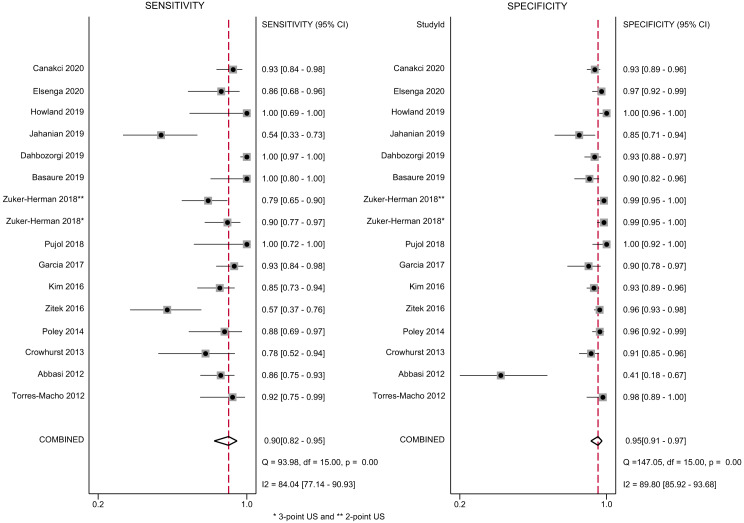
Forest plots of sensitivity and specificity of ultrasound performed by emergency physician for the diagnosis of lower extremities deep vein thrombosis (DVT).

Both the study-specific and pooled sensitivities, specificities, and respective 95% CIs are shown in Figure 2. Compared to the reference standard, the pooled sensitivity and specificity of the EP-performed US for diagnosis of DVT of the lower limb was 90% (95%, CI 82%–95%) and 95% (95%, CI 91%–97%), respectively. I^2^ and Q-test statistics suggested significant heterogeneity between studies (Figure 2). The pooled positive and negative likelihood ratio for the same comparisons were, respectively, 19.1 (95%, CI 10.2–35.8) and 0.10 (95%, CI 0.06–0.19) (data not shown).

### Subgroup Analyses and Meta-Regression

**Figure 3.**
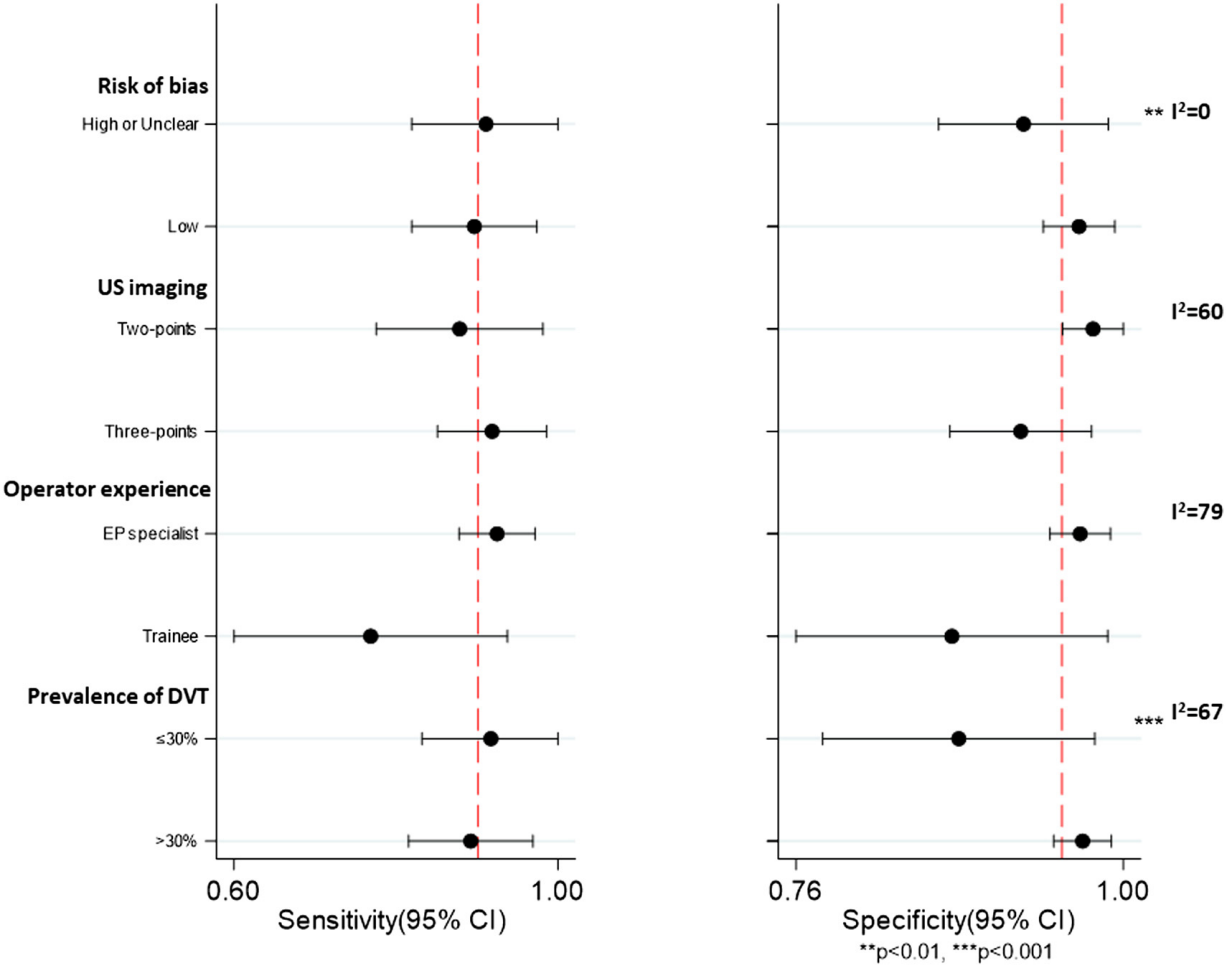
Subgroup analyses for sensitivity and specificity according to selected study characteristics. I^2^ to assess heterogeneity and meta-regression *P*-values for differences in the accuracy within subgroups. The dotted line represents reference values obtained in the pooled sensitivity and specificity of all studies. *US*, ultrasound; *EP*, emergency physician; *DVT*, deep vein thrombosis; *CI*, confidence interval.

We performed exploratory meta-regression analyses with only one explanatory variable added to the model, considering the limited number of studies included. We assessed presence of bias, two-point vs three-point CUS, prior experience of the EP, prevalence of DVT reported (less than or greater than 30%), and sample size. The experience of the EP and increased prevalence of DVT in the sample were found to be significantly associated with improved sensitivity and specificity (meta-regression joint model *P* = 0.01 and 0.05, respectively) (Figure 3). Trainee sensitivity was 77% vs 93% within the attending group. Specificity was 87% and 97%, respectively. The sensitivity of two-point and three-point CUS were 88% and 92%, respectively. When assessing for accuracy this was a non-statistically significant improvement (*P* = 0.07).

Heterogeneity was substantially reduced with respect to the pooled sensitivity and specificity for the studies including only specialist EPs. Given these findings, we performed further subgroup analysis on specialist EP-performed studies. Two-point CUS studies performed by specialist EPs had a pooled sensitivity of 88% compared to the 95% found for three-point CUS also performed by specialist EPs (*P* = 0.02). Specificity of US performed by EP specialist was not different when comparing two- to three-points US.

### Quality Assessment

**Figure 4.**
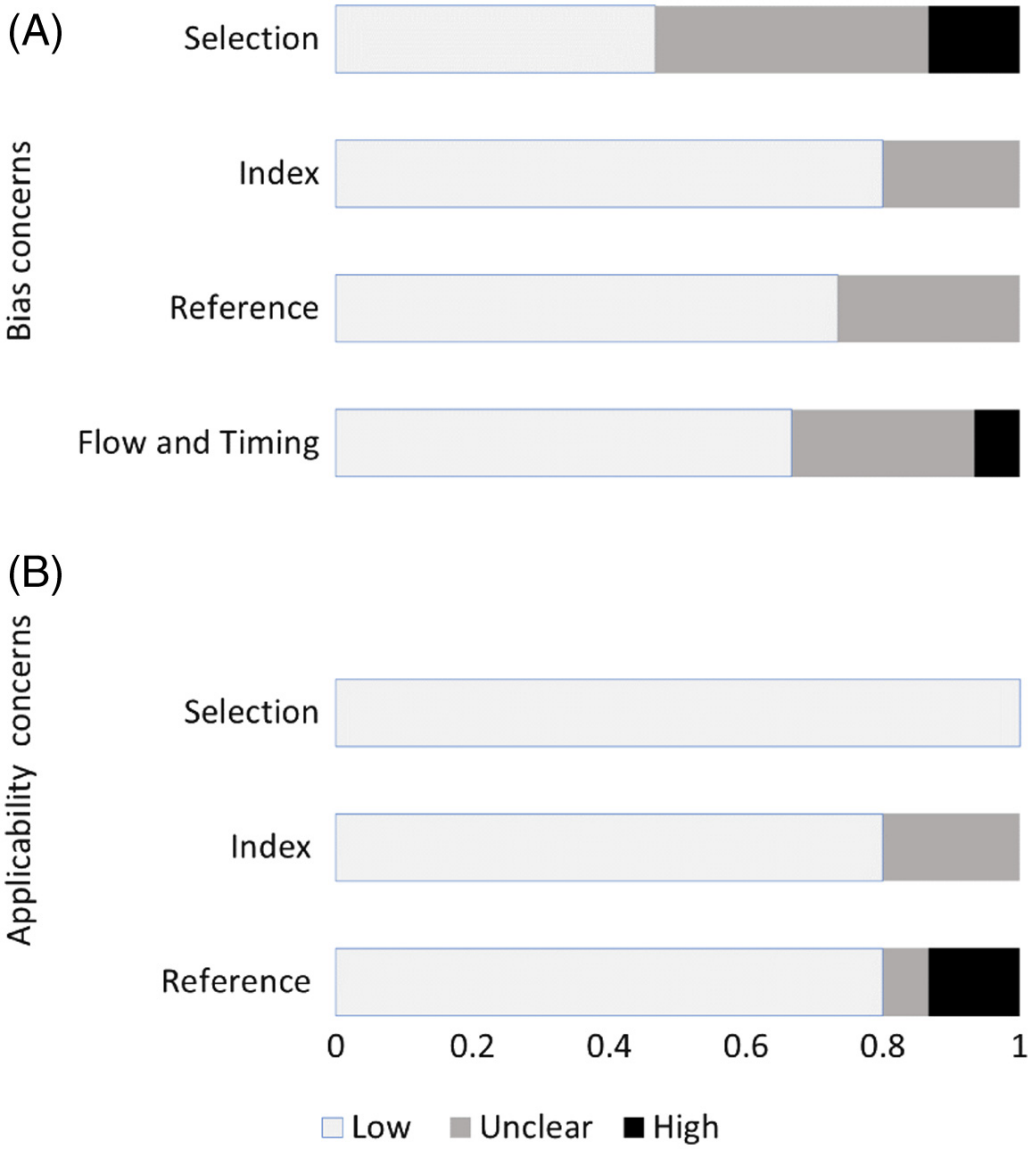
Aggregate assessment of individual study quality according to QUADAS-2 tool.

Based on the QUADAS-2 tool for assessment of the quality of the individual studies, there were concerns regarding the risk of bias (Figure 4). The aggregate risk of bias identified that 40% of studies were considered high or unclear risk of bias of patient selection due to the use of convenience, non-consecutive sampling. Concerns regarding high or unclear risk of biases related to the index test, the reference standard, blinding, or the flow and timing (of the index procedure relative to the reference test) were found in fewer than 30% of the studies included (Figure 4A). The rating of each individual study regarding the QUADAS-2 biases assessed is shown in Supplemental Table 1.

### Sensitivity Analyses

We performed sensitivity analyses excluding studies^16^ ^,^ ^19^ ^,^ ^24^ ^–^ ^27^ that were outliers based on model fitting and outliers’ assessment. Pooled accuracy for the remaining 11 results was slightly lower, and heterogeneity reduced substantially (sensitivity 89%, 95% CI 85%–92%, and I^2^ = 27.8; specificity 96%, 95%, CI 93%–97%, I^2^ = 60.3). Lastly, analyses restricted to studies for which the risk of bias was considered low for all domains yielded similar pooled sensitivity and specificity (data not shown).

### Updated Search

We performed a new literature search in late 2022. Only one new relevant study of 100 patients had been published since June 2021.^30^ An exploratory analysis adding this study to the pool of 16 studies previously assessed showed no differences in the pooled results reported.

## DISCUSSION

The diagnosis of DVT in the ED evolved from cumbersome tests performed outside the ED, such as impedance plethysmography and venography, to easily implemented POCUS that is mainstay training of current EM curriculum in the United States and some other countries.^31^ ^,^ ^32^ Despite widespread use of POCUS, concerns persist regarding the accuracy of tests done in widely disparate EDs. An earlier quantitative systematic review of studies performed in the US yielded sensitivities greater than 95%.^33^ However, as more diverse studies were published, a subsequent review demonstrated a more moderate pooled sensitivity close to 90%.^13^ Both reviews demonstrated very high specificity. Unfortunately, to date all meta-analyses addressing this topic have been plagued by high levels of heterogeneity, a problem identified in a recent review by Lee.^12^ No model has been proposed to reduce heterogeneity.

To our knowledge this is the first systematic review with a focus to maximize performance of ED-based DVT POCUS with recommendations on operator and technique. We identified trends explaining study variability as well as key biases within the literature. In this meta-analysis, using the most recent studies on the use of POCUS in EDs from multiple countries, we demonstrated a pooled sensitivity and specificity of 90% and 95%, respectively. These results are somewhat similar to prior systematic reviews on ED-based DVT POCUS. However, clinically significant variation in operator and scanning protocol existed in the subgroups examined.

General operator level of training (trainee/resident vs attending/fellow/specialist status) was an important predictor of performance with 77% sensitivity noted in the trainee group vs 93% in the specialist group. Specificity in these groups was 87% and 97%, respectively. This is in sharp contrast with training provided as part of the included studies. A quantitative analysis of training immediately pre-intervention was not possible due to lack of detailed information. With what has been reported, its effect on accuracy appears to be far less than general level of training/specialization. Completion of formal EM training pathway appears to have a strong effect on POCUS DVT US performance.

This review spans 10 nations from 2012-2022, representing different approaches to EM and ultrasound training and is, therefore, broadly applicable to contemporary practice. While specialty training is often country-specific,^34^ ^–^ ^36^ most of these countries now include dedicated POCUS training as a mandatory requirement for EM specialist qualification with subspecialist US certification available as well. Ultrasound technique across all included countries tended to be similar, with a reliance on CUS of the proximal leg veins, in accordance with internationally published guidelines on the diagnosis of DVT.^32^

Another unsettled question for the EM application of POCUS for the diagnosis of DVT is whether three-point US is superior compared to the commonly implemented two-point examination. A 2018 radiologist consensus report recommends three-point rather than two-point CUS as a base requirement for diagnosis of DVT because three-point CUS detects isolated femoral vein thromboses that would otherwise be missed in 5%–8% of those with lower extremity DVT.^7^ The study by Adhikari et al,^10^ analyzing three years of radiology-performed CUS in the ED also found that three-point CUS detected an additional 6% of lower extremity DVT isolated to the femoral vein, without involvement of the common femoral vein or popliteal vein. Lastly, the study of Tabbut et al found a similar rate of isolated thrombi from a mix of POCUS and radiology-performed studies.^37^

One of this review’s studies explored the sensitivity of two vs three-point US exams performed by trainees and specialists as a *within-patient* analysis. The sensitivity for the diagnosis of DVT increased by 7% by including the third site. These results are intuitive even in cases of non-isolated femoral vein thrombi. Scanning multiple sites reduces the probability of false negative scans as just a single positive finding is a requirement for diagnosis. Our pooled analysis of two-point vs three-point scanning yielded a 5% higher point estimate of sensitivity for the more comprehensive scan without loss in specificity, which is congruent with prior literature. The difference was not statistically significant with a *P*-value of 0.07. When limited to only specialist-performed exams, the difference was statistically significant (*P* = 0.02).

We found large reductions in heterogeneity in multiple subgroups when looking at studies of attending physician-performed POCUS. These include specialty trained EP-performed two-point and three-point scans and studies without high levels of bias. This implies a higher degree of confidence in the consistency of the intervention’s performance in qualified hands. Subgroup analyses with prevalence below or above 30% yielded increased specificity for studies with prevalence above 30%. However, a 30% prevalence of DVT in the ED is unusually high and unexpected. Differences in patient inclusion criteria (Wells scoring and/or D-dimer) may have contributed to this effect. The potential effect of high prevalence of DVT on the diagnostic accuracy studies is yet to be confirmed.

## LIMITATIONS

This meta-analysis has some limitations. First, because only 15 studies were identified, more complex analyses could not be performed. Furthermore, most studies contained elements of bias, especially related to patient selection; recruitment often occurred as a convenience sample, presumably selected by the ultrasound operator/clinician. Additionally, three studies included inconclusive results.^18^ ^,^ ^20^ ^,^ ^28^ We followed best practices and made the decision to classify inconclusive cases as all positive or all negative depending on the clinical context.^38^ Based on the study design reported by the authors, we categorized the inconclusive results as negative. A sensitivity analysis was conducted, and the limited number of inconclusive results are unlikely to affect the pooled results hereby reported.

Another limitation relates to the inability to better characterize the level of experience of the US operator due to limited detailed information on operator training (Supplemental Table 2). Lastly, restricting publications from 2012 to the present limited the number of studies and the power to assess potential subgroup differences. However, since 2012 formalized training in POCUS has been adopted as part of specialist training in most countries included in this review. Thus, we believe that this review’s results are more generalizable to the broad EM population.

## CONCLUSION

This meta-analysis of studies reported since 2012 demonstrated excellent performance of EM specialist-performed three-point point-of-care ultrasound for the diagnosis of deep vein thrombosis. Both the pooled sensitivity and specificity were 95%. We recommend that POCUS-trained attending EPs perform a three-point examination in the ED to effectively and accurately diagnose DVT. Future general studies on ED-based DVT POCUS are unlikely to modify these findings given the numerous existing studies of at least moderate quality. Future studies of rigorous methodology further addressing certain subgroups are recommended.

## Supplementary Information




