# Supplementary material for: Ultrasound Performed by Emergency Physicians for Deep Vein Thrombosis: A Systematic Review
Source: West J Emerg Med. 2024 Feb 9;25(2):282–90. doi: 10.5811/westjem.18125 (PMC11000565; doi:10.5811/westjem.18125)
Supplement: Supplementary file 1 [file wjem-25-282-s001.docx]

| **Supplemental Table 1.** Assessment of each individual study quality according to QUADAS-2 tool, | | | | | | | | |
| --- | --- | --- | --- | --- | --- | --- | --- | --- |
|  | Risk of bias | | | |  | Applicability | | |
| Author, year | Selection | Index | Reference | Flow and timing |  | Selection | Index | Reference |
| Torres-Macho, 2012 | 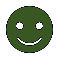 | 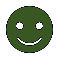 | 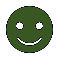 | 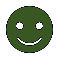 |  | 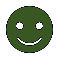 | 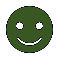 | 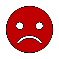 |
| Abbasi, 2012 | 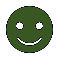 | 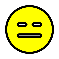 | 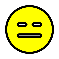 | 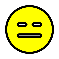 |  | 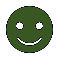 | 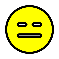 | 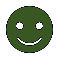 |
| Crowhurst, 2013 | 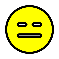 | 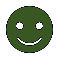 | 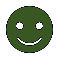 | 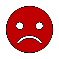 |  | 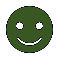 | 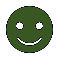 | 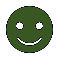 |
| Poley, 2014 | 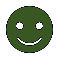 | 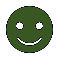 | 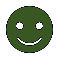 | 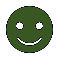 |  | 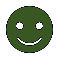 | 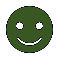 | 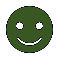 |
| Zitek, 2016 | 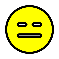 | 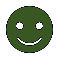 | 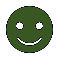 | 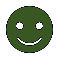 |  | 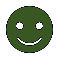 | 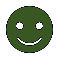 | 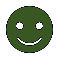 |
| Kim, 2016 | 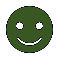 | 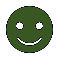 | 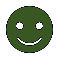 | 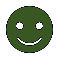 |  | 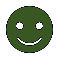 | 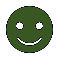 | 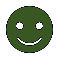 |
| Pedraza-Garcia, 2017 | 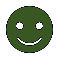 | 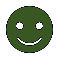 | 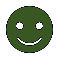 | 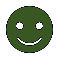 |  | 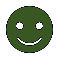 | 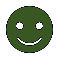 | 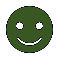 |
| Zuker-Herman, 2018 | 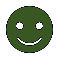 | 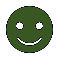 | 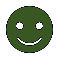 | 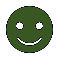 |  | 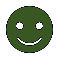 | 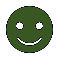 | 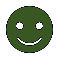 |
| Pujol, 2018 | 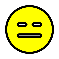 | 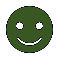 | 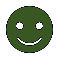 | 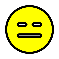 |  | 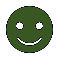 | 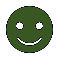 | 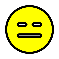 |
| Dehbozorgi, 2019 | 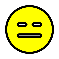 | 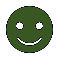 | 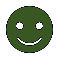 | 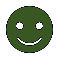 |  | 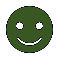 | 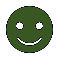 | 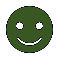 |
| Basaure, 2019 | 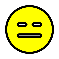 | 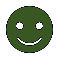 | 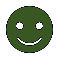 | 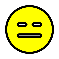 |  | 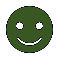 | 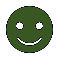 | 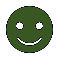 |
| Jahanian, 2019 | 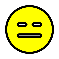 | 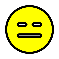 | 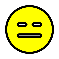 | 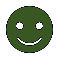 |  | 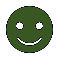 | 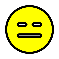 | 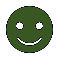 |
| Howland, 2019 | 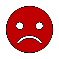 | 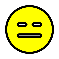 | 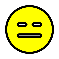 | 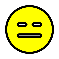 |  | 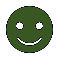 | 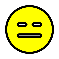 | 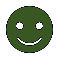 |
| Elsenga, 2020 | 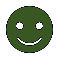 | 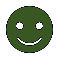 | 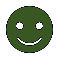 | 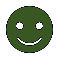 |  | 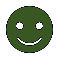 | 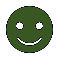 | 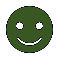 |
| Canakci, 2020 | 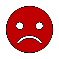 | 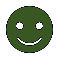 |  |  |  |  |  |  |
| Colored faces represent the risk of bias or concerns with applicability and red=high concern, green=low concern, yellow=unclear. | | | | | | | | |

| Supplemental Table 2. Study-specific description of test-operator training in point-of-care ultrasound for deep vein thrombosis prior to the study. | | | |
| --- | --- | --- | --- |
| Author, year | Patient’s country | Number of tests* | Prior US training received by operator |
| Torres-Macho, 2012 | Spain | 76* | No prior US experience. 2 days course prior to the study |
| Abbasi, 2012 | Iran | 81 | Performed by a 2^nd^-year resident with unclear training with supervision from "Attending physician, who was expert in performing CUS for diagnosing suspected DVT" |
| Crowhurst, 2013 | Australia | 178* | "Emergency medicine consultants with no training in US for LEDVT were approached for participation, with 15 consenting. Participants were required to have completed any US course accredited by the Australasian College of Emergency Medicine and performed at least 20 ultrasonographic examinations for unrelated purposes. Participating EPs completed a standardized 2 h training session ..." |
| Poley, 2014 | Canada | 227 | "A total of 17 physicians (five attending EPs, 12 EM residents) agreed to participate in the study and underwent training. US experience varied widely among the trained physicians, but only three physicians had completed fellowship training in EM US. All physicians had to be considered proficient in US to participate, and each had performed a minimum of 50 bedside US scans in each of the following: aorta, pericardium, focused assessment with sonography in trauma, and obstetrics/gynecology. For this study, a 2-hour didactic training session was given by the authors (RP, JN), followed the next day by a minimum of 12 supervised scans on healthy volunteers. " |
| Zitek, 2016 | United States | 385* | "EM residents in our facility had no previous formal training in the two-point compression technique for diagnosing proximal lower extremity DVTs. All EM residents received approximately a two-hour training session". |
| Kim, 2016 | United States | 296 | "Physician participation in this study was open to all emergency medicine attendings, fellows, and residents, regardless of prior ultrasound experience. LCUS for DVT was a new application at this time, so no EPs had prior experience performing DVT studies. All physicians received a 1-hr didactic lecture and were required to attend at least one practical hands-on session. " |
| Pedraza-Garcia, 2017 | Spain | 109 | All physicians involved had performed more than 30 POCUS for DVT prior to initiation of the protocol during a 6 months "run-in." |
| Zuker-Herman, 2018 | Israel | 195 | "In Israel, EM residents participate in a course of basic ED skills, including the POCUS exam. Some of the residents in the current study participated in this course…  Participants underwent 1 week of ultrasound training with overall 12 h, by an ultrasound specialist radiologist, approved by the Israel Emergency Society." |
| Pujol, 2018 | France | 56 | "… made by an experienced EP (defined as owning an ultrasonography university diploma associated with daily practice). " |
| Dehbozorgi, 2019 | Iran | 240 | "… trained 3rd‐year residents supervised by an ED attending physician who was an Iranian board certified in emergency medicine, a faculty member, and who was an expert in performing 3 PCUS." |
| Basaure, 2019 | Chile | 101 | No data on prior formal training. One pre-study short course |
| Jahanian, 2019 | Iran | 72 | No specific data, but implied no prior DVT POCUS training aside from one pre-study short course. |
| Howland, 2019 | Australia | 100 | Per direct contact with author: Participants were facile ("effectively fellowship trained") in DVT POCUS prior to the study. |
| Elsenga, 2020 | Netherlands | 138 | No data on prior formal training. One pre-study short course. |
| Canakci, 2020 | Turkey | 266 | “All emergency residents and emergency physicians had received DVT US training "in accordance with ACEP recommendations," |

*US*, ultrasound; *CUS,* compression ulttrasonography; *DVT*, deep vein thrombosis; *POCUS*, point-of-care ultrasound; *ED*, emergency department; *EP*, emergency physician; *EM*, emergency medicine; *LEDVT*, lower extremity DVT; *LCUS*, limited compression ultrasound; *ACEP*, American College of Emergency Physicians.

**Appendix 1.** Search terms/strategy

**Ovid MEDLINE 2012-Current (June 28, 2021):**

Search Strategy

1. exp Ultrasonography/ (52300)

2. Femoral Vein/ (1467)

3. exp Venous Thrombosis/ (10621)

4. Thrombophlebitis/ (463)

5. Emergencies/ (8450)

6. Emergency Medicine/ (5223)

7. Emergency Medical Services/ (16120)

8. Emergency Service, Hospital (38369)

9. 1 or 2 (53659)

10. 3 or 4 (11015)

11. 5 or 6 or 7 or 8 (63222)

12. 10 and 11 and 12 (43)

**EMBASE (2012-Current (June 28, 2021):**

Search Strategy

1. B scan/(8437)

2. Color Doppler flowmetry/(14769)

3. Compression ultrasonography/(322)

4. B mode compression technique/(1)

5. Echography/(176691)

6. Doppler echography/(111)

7. Ultrasound/(312608)

8. Deep vein thrombosis/(40735)

9. Leg thrombosis/(892)

10. Thrombophlebitis/(3727)

11. Emergency medicine/(129863)

12. Emergency ward/(123638)

13. Emergency health service/(51761)

14. #1 or #2 or # 3 or #4 or #5 or #6 or #7/(422430)

15. #8 or #9 or #10/(44056)

16. #11 or #12 or #13/(252304)

17. #14 and #15/(6656)

18. #16 and #17/(649)

19. #18 and ('clinical study'/de OR 'clinical trial'/de OR 'clinical trial topic'/de OR 'cohort

analysis'/de OR 'controlled clinical trial'/de OR 'controlled study'/de OR 'cross

sectional study'/de OR 'diagnostic test accuracy study'/de OR 'observational

study'/de OR 'prospective study'/de OR 'randomized controlled trial'/de

OR 'randomized controlled trial topic'/de)/(200)
